# FGF23/FGFR4-mediated left ventricular hypertrophy is reversible

**DOI:** 10.1038/s41598-017-02068-6

**Published:** 2017-05-16

**Authors:** Alexander Grabner, Karla Schramm, Neerupma Silswal, Matt Hendrix, Christopher Yanucil, Brian Czaya, Saurav Singh, Myles Wolf, Sven Hermann, Jörg Stypmann, Giovana Seno Di Marco, Marcus Brand, Michael J. Wacker, Christian Faul

**Affiliations:** 1 Katz Family Drug Discovery Center and Division of Nephrology and Hypertension, Department of Medicine, University of Miami Miller School of Medicine, Miami, Florida USA; 2 Division of Nephrology, Department of Medicine, Duke University Medical Center, Duke University, Durham, North Carolina USA; 3 Department of Cell Biology and Anatomy, University of Miami Miller School of Medicine, Miami, Florida USA; 4 Department of Biomedical Sciences, School of Medicine, University of Missouri-Kansas City, Kansas City, Missouri USA; 5 Division of Nephrology, Department of Medicine, The University of Alabama at Birmingham, Birmingham, Alabama USA; 6 European Institute for Molecular Imaging, University of Münster, Münster, Germany; 7 Department of Cardiovascular Medicine, University Hospital Münster, Münster, Germany; 8 Department of Internal Medicine D, University Hospital Münster, Münster, Germany

## Abstract

Fibroblast growth factor (FGF) 23 is a phosphaturic hormone that directly targets cardiac myocytes via FGF receptor (FGFR) 4 thereby inducing hypertrophic myocyte growth and the development of left ventricular hypertrophy (LVH) in rodents. Serum FGF23 levels are highly elevated in patients with chronic kidney disease (CKD), and it is likely that FGF23 directly contributes to the high rates of LVH and cardiac death in CKD. It is currently unknown if the cardiac effects of FGF23 are solely pathological, or if they potentially can be reversed. Here, we report that FGF23-induced cardiac hypertrophy is reversible *in vitro* and *in vivo* upon removal of the hypertrophic stimulus. Specific blockade of FGFR4 attenuates established LVH in the 5/6 nephrectomy rat model of CKD. Since CKD mimics a form of accelerated cardiovascular aging, we also studied age-related cardiac remodeling. We show that aging mice lacking FGFR4 are protected from LVH. Finally, FGF23 increases cardiac contractility via FGFR4, while known effects of FGF23 on aortic relaxation do not require FGFR4. Taken together, our data highlight a role of FGF23/FGFR4 signaling in the regulation of cardiac remodeling and function, and indicate that pharmacological interference with cardiac FGF23/FGFR4 signaling might protect from CKD- and age-related LVH.

## Introduction

Left ventricular hypertrophy (LVH) is one of the major cardiovascular complications in patients with chronic kidney disease (CKD) that contributes to diastolic dysfunction, congestive heart failure, arrhythmias and death^1^. The complex pathomechanism of LVH in CKD comprises traditional risk factors such as ventricular pressure overload, volume overload, hypertension and anemia^2, 3^, but also includes recently emerging signaling pathways involving fibroblast growth factors (FGF) and their cell surface receptors^4^. FGF23 is one of the master regulators of serum phosphate and calcium homeostasis^5^. In patients with CKD, circulating FGF23 levels gradually rise with the decline of kidney function, which maintains normal serum phosphate levels as the renal capability for phosphate excretion decreases^6^.

Several large epidemiological studies have demonstrated strong dose-dependent associations between elevated serum FGF23 levels and increased risk of cardiovascular events and mortality in CKD patients^7–9^. Elevated FGF23 independently correlates with greater left ventricular mass and increased incidence and prevalence of LVH^10^. In recent translational studies, we demonstrated that FGF23 directly induces hypertrophic growth of cardiac myocytes *in vitro* and LVH in rodents^10–12^. The hypertrophic effects of FGF23 are mediated by activation of cardiac FGF receptor 4 (FGFR4)^11^, resulting in the recruitment and phosphorylation of phospholipase Cγ (PLCγ) and the subsequent activation of the calcineurin/nuclear factor of activated T cells (NFAT) signaling cascade, which is a potent inducer of ventricular remodeling in response to different pathogenic stimuli^13, 14^. We have also demonstrated that FGF23 elevates intracellular calcium levels in isolated cardiac myocytes and increases contractility in mouse ventricular muscle strips *ex vivo* ^12^.

Here, we determine if FGFR4 mediates acute effects of FGF23 on cardiac contractility. Furthermore, we study whether lowering FGF23 concentrations can reduce established cardiac hypertrophy, which has been induced by FGF23 elevation *in vitro* and *in vivo*. We also test if treatment of the 5/6 nephrectomy rat model of CKD with an FGFR4-specific blocking antibody could reverse established LVH. Finally, as CKD and aging share several similarities in regards to cardiovascular alterations^15–17^, including increased prevalence of LVH^18^, and serum FGF23 levels are also elevated in the non-CKD elderly population^8, 19, 20^, we determine if FGFR4 is required for aging-related LVH in mice.

Currently, it is still unknown whether FGF23/FGFR4-induced cardiac remodeling does inevitably lead to pathological alterations and heart failure, or if initially the activation of this signaling mechanism has physiologically relevant effects that are beneficial for cardiac function. We postulate that if FGF23’s actions on cardiac myocytes are reversible, the pharmacological blockade of FGF23/FGFR4 signaling might attenuate LVH and prevent the progression of cardiac injury in CKD.

## Results

### FGF23 increases ventricular contraction via FGFR4

**Figure 1:**
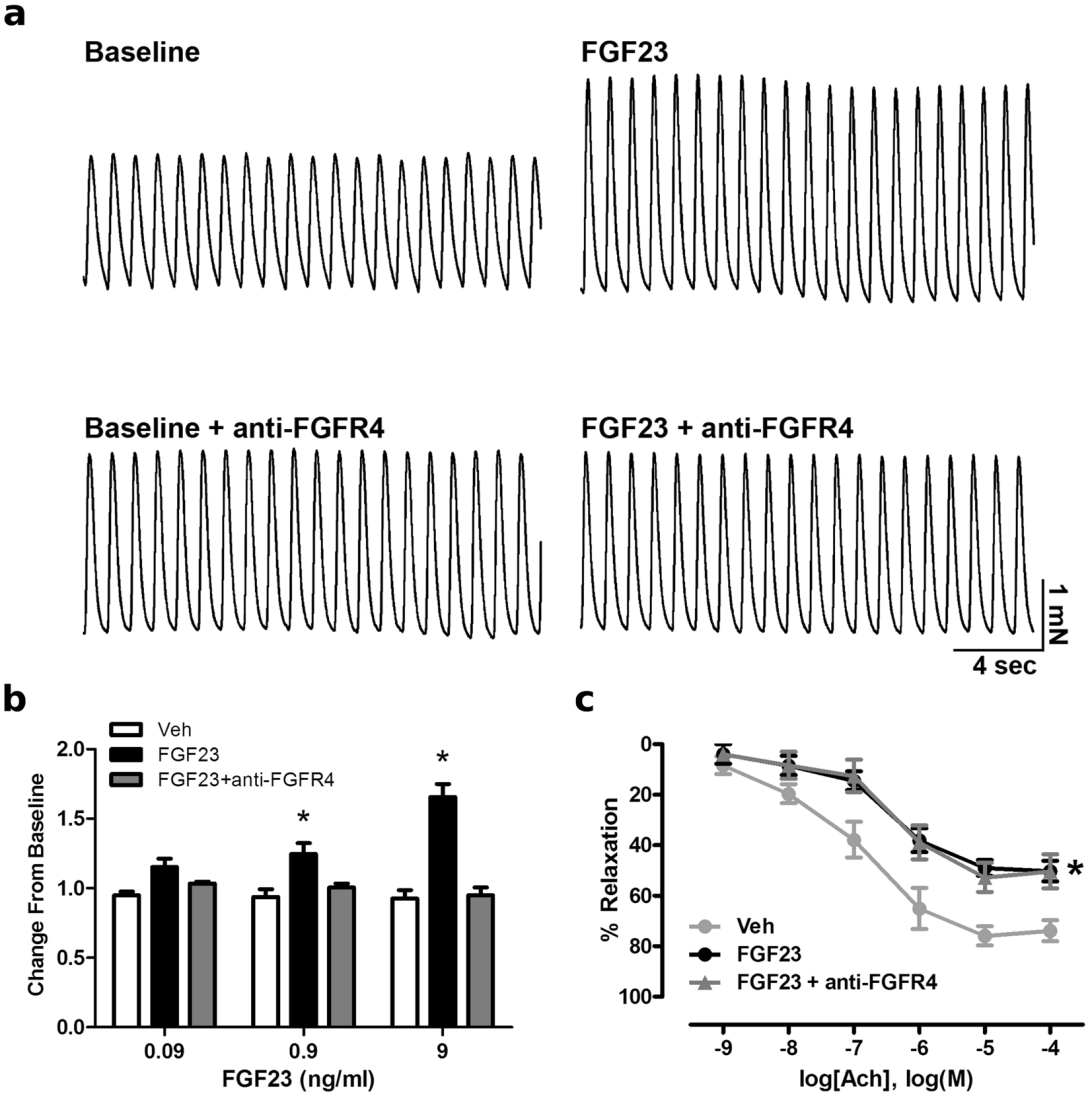
FGF23 increases ventricular contraction force via FGFR4. (**a**) Raw tracings of 1 Hz paced left ventricular muscle strip contractions at baseline and 20 min following treatment with 9 ng/ml FGF23 alone or in the presence of an FGFR4-specific blocking antibody (anti-FGFR4) at 10.5 µg/ml. (**b**) Mean changes in isometric tension data induced by vehicle and FGF23 (0.09–9 ng/ml). FGF23 increases mean isometric tension compared to vehicle which is inhibited by pretreatment with anti-FGFR4 pretreatment at 10.5 µg/ml. (**c**) Mouse aortic rings were treated with FGF23 (9 ng/ml) prior to preconstriction with PGF_2α_ (10 µM) and concentration-response relaxation curves to acetylcholine (Ach) were determined. Pretreatment of aortic rings with anti-FGFR4 for 30 min does not prevent the FGF23-mediated impairment of relaxation. Data are shown as mean ± SE (n = 5; *p < 0.05 compared to vehicle).

To determine if FGF23’s effect on ventricular contractility is mediated specifically by the FGFR4 isoform, we analyzed the contractile responses of cardiac muscle strips isolated from mice elicited by increasing concentrations of FGF23 following pre-treatment with a FGFR4-specific blocking antibody (anti-FGFR4)^11^. Increasing concentrations of FGF23 elevated isometric force when compared to vehicle, similar to our previous study^12^, while pre-treatment of muscle strips with anti-FGFR4 eliminated this effect of FGF23 (Fig. 1a). In addition, we also analyzed slope, area under the curve, and time of relaxation (t) of the contraction waveforms. Comparing the change from its own baseline for treatments of vehicle, FGF23 (9 ng/ml) and FGF23 with anti-FGFR4 (10.5 µg/ml) pre-treatment, respectively the slope was 0.98 ± 0.06, 1.81 ± 0.12*, 0.94 ± 0.06; area was 0.86 ± 0.05, 1.26 ± 0.11*, 0.89 ± 0.07; and t was 0.97 ± 0.05, 0.86 ± 0.03, 1.03 ± 0.04 (*statistical difference from vehicle; P < 0.05) (Fig. 1b).

We have previously demonstrated that FGF23 increases superoxide levels and reduces nitric oxide bioavailability in endothelial cells and impairs relaxation of isolated mouse aortic rings which is blocked in the presence of a pan-FGFR inhibitor^21^. To determine if FGF23’s effect on aortic relaxation requires FGFR4, we pre-treated aortic rings with anti-FGFR4 followed by FGF23 treatment at 9 ng/ml which did not improve relaxation (Fig. 1c), indicating that other FGFR isoforms than FGFR4 are involved. Combined, these data demonstrate that FGF23 increases cardiac contractility via FGFR4, which we have shown before to also mediate FGF23-induced hypertrophic growth of cardiac myocytes^11^, and suggest that FGF23 utilizes different FGFR isoforms to directly target the heart and aorta.

### FGF23-induced hypertrophy in cultured cardiac myocytes is reversible

**Figure 2:**
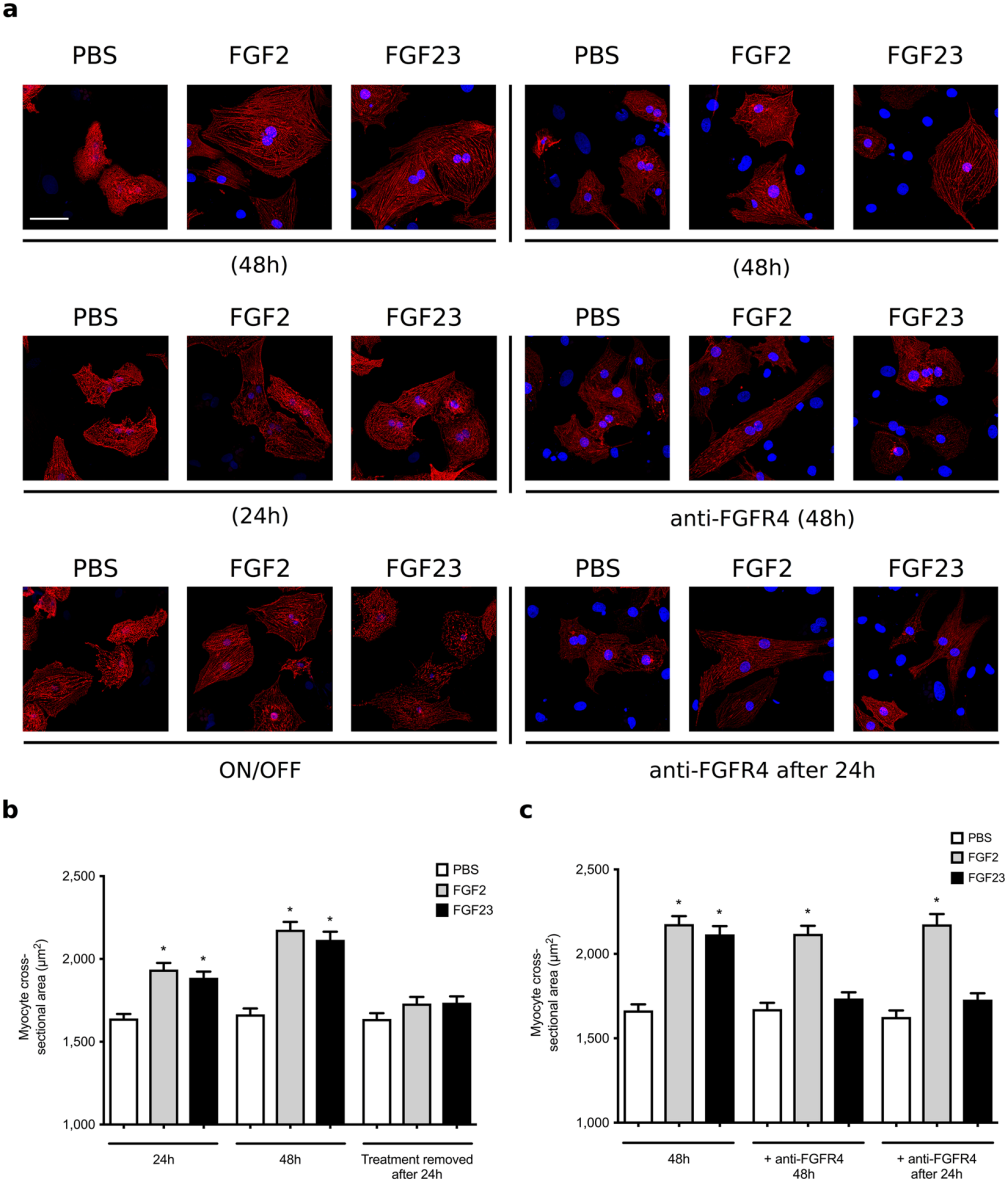
FGF23-induced hypertrophic growth of cultured cardiac myocytes is reversible. Analysis of hypertrophic cell growth in isolated neonatal rat ventricular myocytes (NRVM). Cells were treated with recombinant FGF23 or FGF2 at 25 ng/ml or with solvent (PBS) for various time periods. A subset of cells was co-treated with anti-FGFR4 at 10 µg/ml. Cross-sectional cell area was determined by confocal fluorescence microscopy based on α-actinin-positive immunostaining. (**a**) Representative immunofluorescence confocal images of isolated NRVMs. Myocytes are labeled with anti-α-actinin (red), and DAPI (blue) identifies nuclei (original magnification x63, scale bar 50 µm). (**b**) Within 24 and 48 hours of treatment, FGF23 and FGF2 significantly increase myocyte area. When NRVM are treated with FGFs for 24 hours, followed by FGF removal for 24 hours, cell area reverse back to levels of PBS-treated control cells. (**c**) When initiated at the same time, co-treatment with anti-FGFR4 blocks the development of FGF23-induced hypertrophy as analyzed after 48 hours (middle panel). When NRVM are treated with FGF23 for 24 hours and then co-treated with FGF23 and anti-FGFR4 for an additional 24 hours, myocyte area reverses back to control levels (right panel). Anti-FGFR4 co-treatment does not affect FGF2-induced hypertrophy. (150 cells per condition; n = 3 independent isolations of NRVM; *p < 0.05 compared with PBS).

FGF23, as well as paracrine FGF2, induce hypertrophic growth of isolated neonatal rat ventricular myocytes (NRVM) within 48 hours^10^. To determine if this effect is reversible, we treated NRVM with FGF23 or FGF2 for 24 hours, followed by incubation in regular culture medium for additional 24 hours. Both FGFs induced hypertrophy after 24 hours with a more prominent effect after 48 hours. Upon removal of the hypertrophic stimulus after 24 hours, NRVM recovered from cellular hypertrophy within 24 hours and did not exhibit significant changes in cross-sectional area when compared to PBS-treated cells (Fig. 2a,b).

We have previously shown that pre-treatment with anti-FGFR4 blocks the development of FGF23-induced hypertrophic growth in NRVM^11^. To test if anti-FGFR4 can reverse FGF23-mediated hypertrophy, NRVM were co-treated with anti-FGFR4 starting 24 hours after initiation of FGF23 treatment, and cells were analyzed 24 hours later. Pre-treatment with anti-FGFR4 blocked FGF23- but not FGF2-mediated hypertrophy (Fig. 2a,c). In addition, treatment with anti-FGFR4 after 24 hours reduced pre-existing cellular hypertrophy to baseline levels, which were not significantly different from PBS-treated cells. These data indicate that FGF23-induced hypertrophic growth of NRVM can be reversed by FGFR4 blockade.

### FGF23-induced LVH in mice is reversible

**Figure 3:**
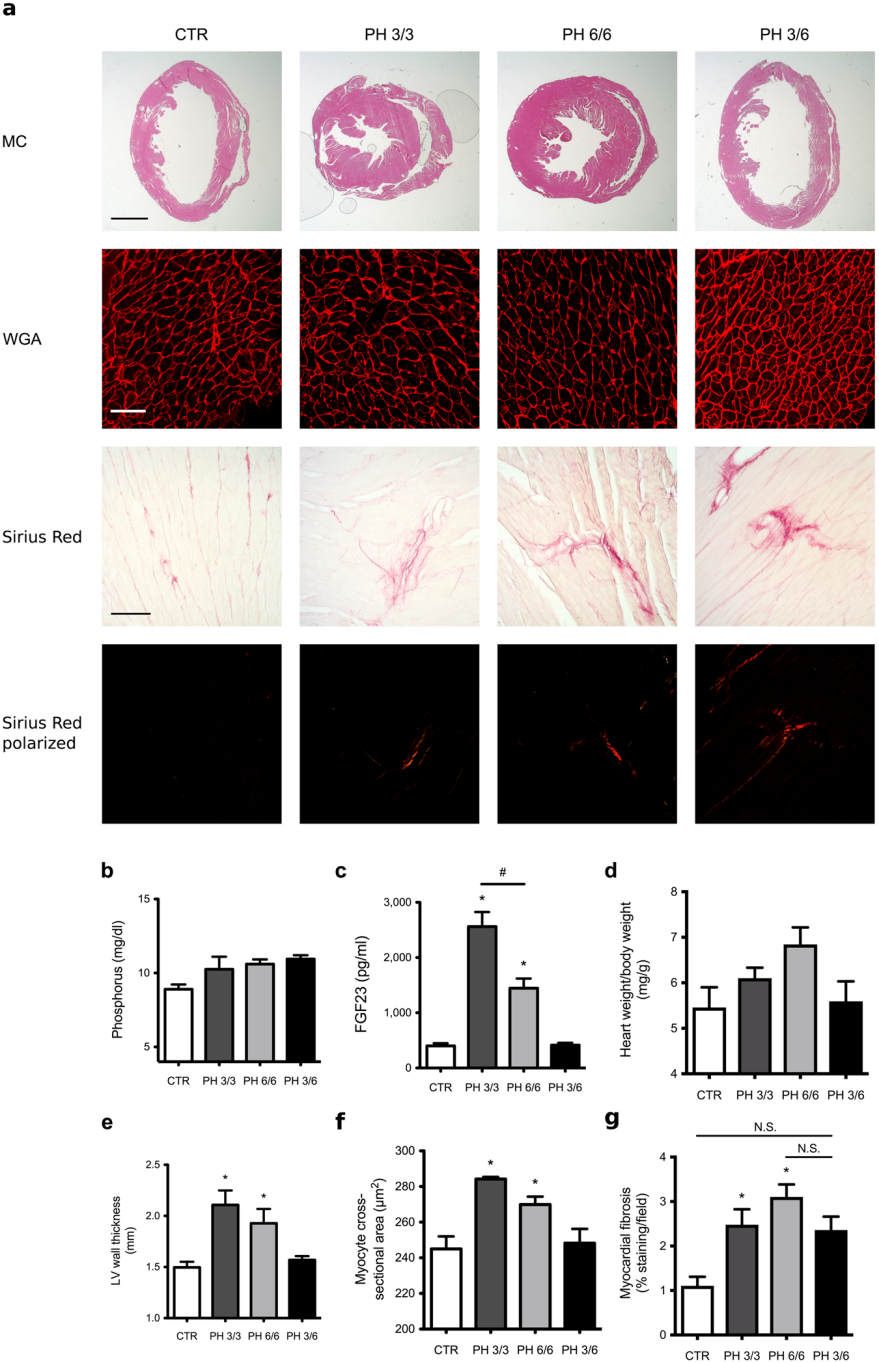
LVH induced by high phosphate diet is reversible in mice. Compared to normal chow (CTR), mice on a high phosphate diet for 3 months (PH 3/3) and 6 months (PH 6/6) develop LVH. Mice on a high phosphate diet for three months followed by a normal chow diet for three months (PH 3/6) do not develop LVH. (**a**) Upper panel: representative gross pathology of mid-chamber (MC) sections of the heart (H&E stain, original magnification x5, scale bar 2 mm). Middle panel: representative WGA-stainings of the LV free wall (original magnification x63, scale bar 50 µm). Lower two panels: representative Picrosirius Red stainings demonstrating fibrosis in the LV free wall (original magnification x10, scale bar 100 µm). (**b**) Compared to CTR, mice on a high phosphate diet (PH 3/3, PH 6/6 and PH 3/6) show a trend towards higher serum phosphate levels. (**c**) Serum FGF23 levels are significantly increased in animals fed a high phosphate diet (PH 3/3, PH 6/6) and return to normal levels when mice are transitioned to normal chow for three months (PH 3/6). (**d**) Mice on high phosphate diet exhibit a trend towards increased ratios of heart weight to body weight. (**e**) Average LV wall thickness as measured by seven individual angles at 0, 30, 60, 90, 120, 150, 180 degrees along the LV free wall from the hemi-circle of the short axis. (**f**) Cross-sectional area of 25 myocytes per field of 4 fields along the LV free wall. (**g**) Quantification of myocardial fibrosis (8 fields of view around the LV). All values are mean ± SEM (n = 4–9 per group; *p < 0.05 compared with CTR, ^#^p < 0.01 between groups).

To determine if FGF23-induced cardiac hypertrophy is reversible *in vivo*, we elevated serum FGF23 levels in C57Bl/6J mice by administration of a 2% phosphate diet for 3 months, that has been shown to induce LVH^11^, as well as for 6 months. Another group of mice was fed a high phosphate diet for 3 months followed by 3 months of regular chow. All animals on high phosphate diet demonstrated slightly elevated serum phosphorus levels (Fig. 3b). Circulating levels of FGF23 were significantly increased in mice on high phosphate diet that reversed to control levels upon switching to regular chow (Fig. 3c). In response to high phosphate diet for 3 and 6 months, mice developed marked LVH, characterized by increased left ventricular wall thickness and increased cross-sectional area of individual cardiac myocytes, when compared to controls (Fig. 3a,d–f). In contrast, animals that were fed a regular diet for 3 months after 3 months of high phosphate diet recovered from LVH and did not exhibit significant changes in left ventricular wall thickness or cardiac myocyte cross-sectional area in comparison to mice that were always on a normal diet (Fig. 3a,d–f). Interestingly, high phosphate diet for 3 and 6 months induced significant myocardial fibrosis, which did not completely recover in animals that were switched to regular chow for an additional 3 months. These animals exhibited cardiac collagen deposition that was not significantly different from mice that were continuously fed a high phosphate diet for 6 months, but also not significantly increased when compared to controls (Fig. 3a,g). Of note, animals on high phosphate diet for 6 months exhibited significantly lower FGF23 levels than animals that were fed the diet for 3 months (Fig. 3c), which was also accompanied by a slight reduction of cardiac hypertrophy that missed statistical significance (Fig. 3a,e,f), but not of cardiac fibrosis (Fig. 3a,g).

These findings indicate that the induction of LVH in mice by high phosphate diet is reversible. Since phosphate elevates serum FGF23 and we have shown before that constitutive FGFR4 knockout mice (FGFR4^−/−^) are protected from phosphate diet-induced LVH^11^, we conclude that FGF23/FGFR4 signaling might play a causative role in the development of reversible LVH in this mouse model.

### FGFR4 blockade inhibits the progression of LVH in the 5/6 nephrectomy rat model of CKD

**Figure 4:**
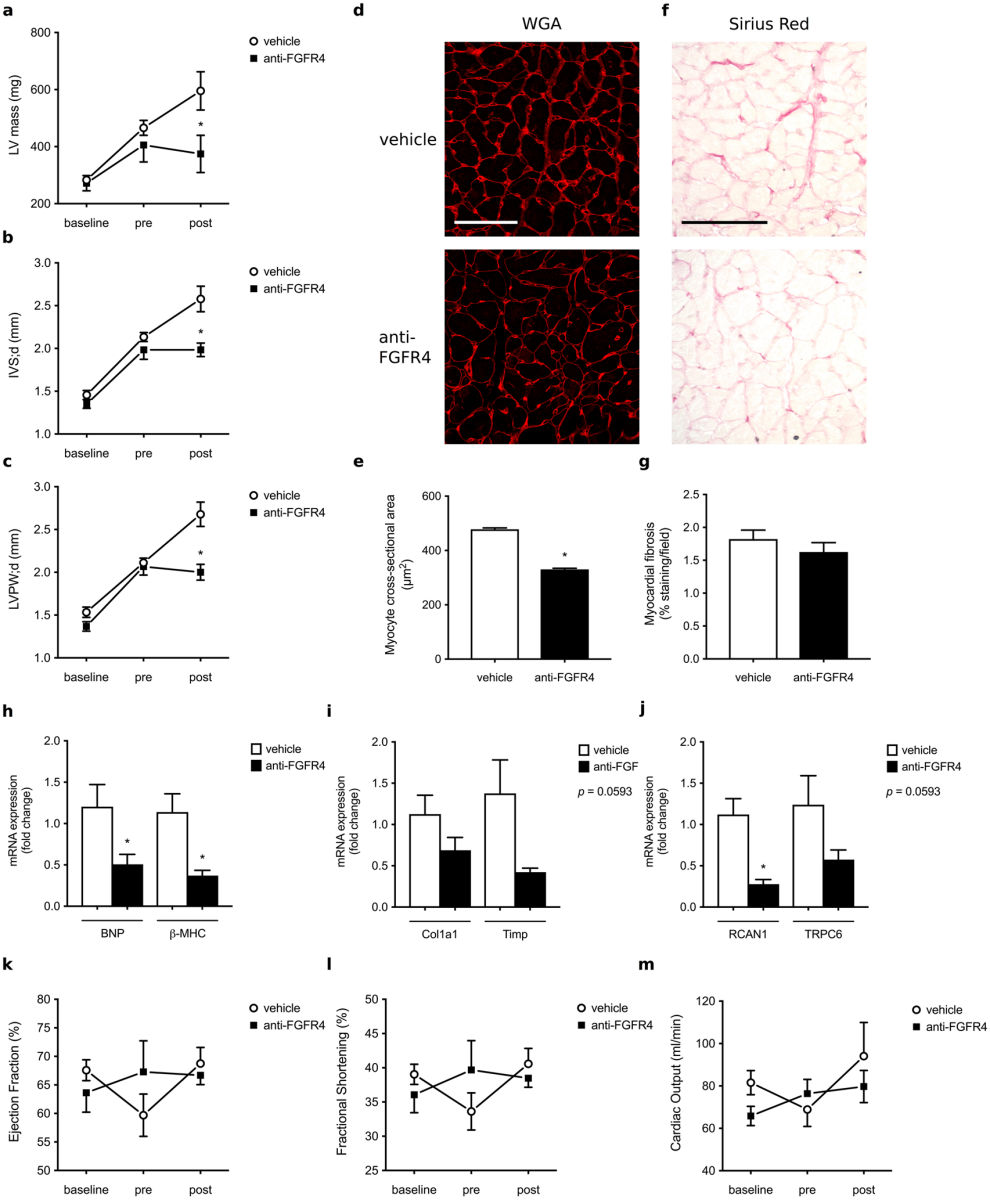
FGFR4 blockade inhibits the progression of LVH in the 5/6 nephrectomy rat model of CKD. (**a**–**c**) Cardiac parameters of 5/6 nephrectomized rats were evaluated by echocardiography at baseline, four weeks (pre) and eight weeks after surgery (post). All 5/6 nephrectomized animals develop LVH four weeks post-surgery, but anti-FGFR4 treatment prevents the progression of cardiac remodeling when compared to vehicle-treated rats. (**d**) Representative images of WGA-stainings of the LV free wall (original magnification x63, scale bar 70 µm). (**e**) Cross-sectional area of 25 myocytes per field of 4 fields along the LV free wall. (**f**) Representative Picrosirius Red stainings of the LV free wall (original magnification x20, scale bar 100 µm). (**g**) Quantification of myocardial fibrosis based on positive Picrosirius Red labeling (8 fields of view around the LV). (**h**,**i**,**j**) Quantitative real-time PCR analysis of cardiac tissue using GAPDH as a house keeping gene. mRNA levels of hypertrophic markers (BNP, β-MHC) and the NFAT target gene RCAN1 are significantly decreased in anti-FGFR4 treated rats when compared to vehicle-injected animals. Fibrosis markers (Col1a1, Timp) and the NFAT target gene TRPC6 show a trend towards a decrease upon FGFR4 blockade (p = 0.0593). (**k**,**l**,**m**) Cardiac function remains unchanged upon induction of CKD as well as before and after administration of anti-FGFR4. No significant differences were found in ejection fraction, fractional shortening and cardiac output over time. All values are mean ± SEM (n = 6–8 per group; *p < 0.05).

We have previously shown that administration of anti-FGFR4 prevents the development of LVH in the 5/6 nephrectomy rat model of CKD^11^, indicating that FGF23/FGFR4 is important for the initiation of cardiac hypertrophy in the context of kidney injury. Furthermore, delivery of a pan-FGFR inhibitor in 5/6 nephrectomized rats with LVH initiated two weeks after surgery improves cardiac structure and function within three weeks^22^. To test whether FGFR4 inhibition also affects previously established LVH, we injected 5/6 nephrectomized rats with anti-FGFR4 starting four weeks after surgery for an additional four weeks. Analyses of renal and metabolic parameters as well as blood pressure and echocardiography were performed one week before surgery (baseline), four weeks after surgery (pre) and four weeks after initiation of treatment (post). All 5/6 nephrectomized animals demonstrated significant reduction in kidney function, hypertension, proteinuria and elevated serum FGF23 levels, with no significant differences between vehicle- and anti-FGFR4-treated animals, as summarized in Table 1. In comparison to baseline measurements, all 5/6 nephrectomized rats developed LVH four weeks after surgery as indicated by elevated left ventricular mass, greater interventricular septal thickness (IVS; d) and increased left ventricular posterior wall thickness (LVPW; d) (Fig. 4a–c). Four weeks of FGFR4 blockade completely prevented the progression of LVH, whereas vehicle-treated animals experienced a further 28% increase in left ventricular mass (Fig. 4a). Anti-FGFR4 treatments significantly decreased myocyte cross-sectional area when compared to vehicle treated animals (Fig. 4d,e). Picrosirius Red stainings revealed no significant differences in cardiac collagen deposition in anti-FGFR4 treated rats when compared to vehicle treated animals (Fig. 4f,g).

**Table 1:** Pharmacological inhibition of FGFR4 does not improve kidney function or blood pressure in 5/6 nephrectomized rats. Analyses of renal and metabolic parameters as well as blood pressure in 5/6 nephrectomized (Nx) rats that were performed one week before surgery (baseline), four weeks after surgery (pre) and four weeks after initiation of treatment (post). Compared with 5/6Nx rats receiving vehicle solution, impaired renal function, hypertension, and elevated serum FGF23 levels are not significantly changed by delivery of a FGFR4-specific blocking antibody (anti-FGFR4). Values are reported as mean ± SEM; *p < 0.05 when compared to baseline 5/6Nx + vehicle; ^#^p < 0.05 when compared to baseline 5/6Nx + anti-FGFR4.

		5/6Nx + vehicle	5/6Nx + anti-FGFR4
Creatinine clearance (ml/min/100 g)	baseline	0.79 ± 0.03	0.95 ± 0.02
	pre	0.28 ± 0.04*	0.32 ± 0.04^#^
	post	0.25 ± 0.04*	0.31 ± 0.05^#^
Blood urea nitrogen (mg/dl)	baseline	12 ± 0.8	15.7 ± 1.1
	pre	39.2 ± 3.8*	34.3 ± 3.5^#^
	post	39.3 ± 3.3*	37.5 ± 5.6^#^
Systolic blood pressure (mmHg)	baseline	141.7 ± 2.9	141.7 ± 5.5
	pre	206.7 ± 6.3*	216.7 ± 5.0^#^
	post	209.1 ± 5.5*	206.8 ± 7.5^#^
Diastolic blood pressure (mmHg)	baseline	100.3 ± 3.7	98.8 ± 4.9
	pre	150.6 ± 11.4*	166.8 ± 5.4^#^
	post	153.2 ± 10.6*	160 ± 9.3^#^
Urine protein (mg/mg creatinine)	baseline	2.79 ± 0.28	2.28 ± 0.28
	pre	15.12 ± 4.72*	13.52 ± 4.38^#^
	post	20.34 ± 5.75*	15.55 ± 4.78^#^
Phosphate (mg/dl)	post	5.94 ± 0.49	7.22 ± 0.58
FGF23 (pg/ml)	post	333 ± 119	485 ± 133

Gene expression analysis demonstrated reduced mRNA levels of brain natriuretic peptide (BNP) and beta-myosin heavy chain (β-MHC) in response to anti-FGFR4 treatment, consistent with the inhibition of fetal gene programs associated with pathological cardiac remodeling (Fig. 4h). Furthermore, quantitative real-time PCR showed a tendency towards a decrease in cardiac expression of fibrosis markers, collagen 1a1 (Col1a1) and tissue inhibitor of matrix metalloproteinases (Timp). However, this decrease missed statistical significance with a p-value of 0.0593 (Fig. 4i). Finally, anti-FGFR4 treatment significantly reduced mRNA levels of established NFAT target genes, such as regulator of calcineurin 1 (RCAN1) and induced a trend towards a reduction of transient receptor potential channel 6 (TRPC6) (p-value of 0.0593; Fig. 4j). Ultimately pathologic cardiac remodeling results in cardiac dysfunction leading to progressive heart failure. However, in this particular model we did not observe significant changes in ejection fraction, fractional shorting and cardiac output over time, indicating that neither the induction of CKD by 5/6 nephrectomy nor anti-FGFR4 treatment affected cardiac function (Fig. 4k–m). Taken together, these findings indicate that specific FGFR4 blockade inhibits the further progression of pathologic cardiac remodeling in 5/6 nephrectomized rats with previously established LVH.

### Mice lacking FGFR4 are protected from age-related LVH

**Figure 5:**
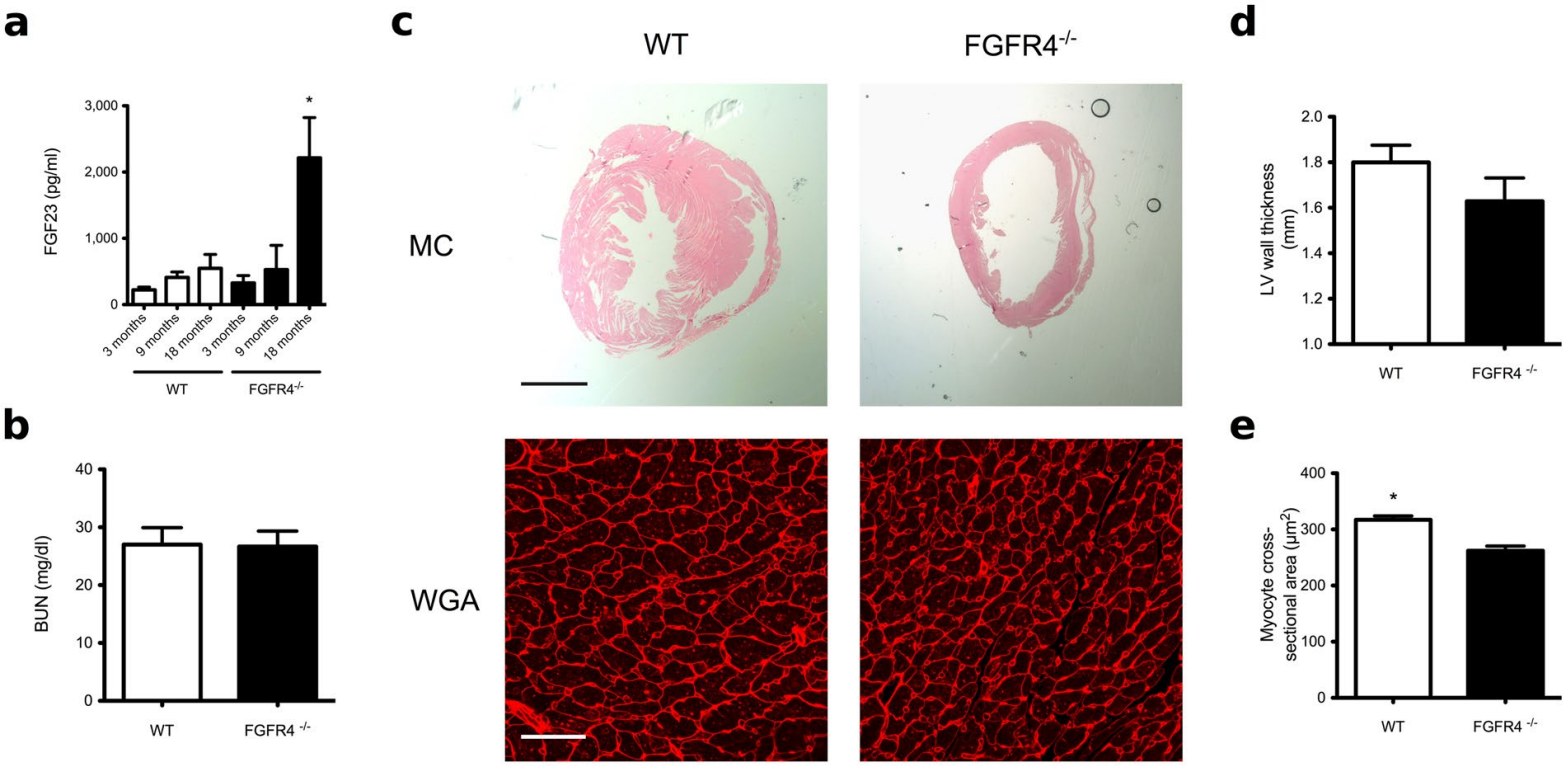
Aging-related LVH in mice requires FGFR4. (**a**) Serum levels of C-terminal FGF23 rise progressively in wild-type (WT) and in global FGFR4 knockout (FGFR4^−/−^) mice. At 18 months of age, FGF23 is significantly elevated in FGFR4^−/−^ mice when compared to WT littermates (n = 5–6 animals per group; *p < 0.05). (**b**) Blood urea nitrogen (BUN) levels are not significantly different between WT and FGFR4^−/−^ mice at 18 months. (**c**) Cardiac analysis of FGFR4^−/−^ and WT littermates at 18 months of age. Depicted are representative gross pathology mid chamber (MC) sections (scale bar 2 mm) and WGA-stained sections from the left ventricular mid-chamber free wall (scale bar 50 µm). (**d**) WT mice exhibit a tendency towards increased left ventricular (LV) wall thickness. (**e**) WT mice manifest significant increases in cross-sectional area of individual myocytes (n = 100 cells/animal: 5–6 animals per group; *p < 0.001).

CKD and aging share several similarities in regards to physiological and cellular alterations, including increased prevalence of cardiovascular injury such as LVH^4, 18^. Human studies have shown that serum FGF23 levels increase with age^20^, and that elevated serum FGF23 levels associate with increased left ventricular mass in elderly subjects^8, 19^. To determine if FGF23/FGFR4 signaling is involved in aging-related LVH, we analyzed the cardiac phenotype of 18-month old FGFR4^−/−^ and wild-type mice. We have shown before that 6-month old FGFR4^−/−^ mice are protected from high phosphate diet-induced LVH, but under normal conditions do not show significant cardiac alterations in comparison to wild-type littermates^11^. Here we found that with increasing age, serum FGF23 levels tend to increase in wild-type mice (Fig. 5a), whereas kidney function was not altered (Fig. 5b). Compared to 6 months of age^11^, 18-month old wild-type mice developed LVH as evident by a trend towards increased left ventricular wall thickness and significantly increased myocyte cross-sectional area (Fig. 5c–e). Although we detected an increase in serum FGF23 levels in 18-month old FGFR4^−/−^ mice that was even higher than in wild-type littermates, FGFR4^−/−^ mice did not develop LVH (Fig. 5c–e). We conclude that FGFR4 is required for the development of LVH in aging mice with high FGF23 levels and normal kidney function.

## Discussion

The present study was designed to advance our understanding of the role of FGF23/FGFR4 signaling in the regulation of cardiac function and remodeling. We found that hypertrophic effects of FGF23 on cardiac myocytes are reversible *in vitro* and *in vivo*, and that an isoform-specific FGFR4 blocking antibody is capable of reversing FGF23-mediated hypertrophic growth of isolated cardiac myocytes and alleviating established LVH in an animal model of CKD with high FGF23 levels. Furthermore, we demonstrate that FGFR4 blockade mitigates the acute FGF23-induced increase in contractility of murine ventricular muscle strips, while FGF23 effects on aortic relaxation are not affected. Finally, mice lacking FGFR4 are protected from age-related cardiac remodeling despite exhibiting significantly increased serum FGF23 levels when compared to wild-type littermates.

Since LVH affects up to 70% of patients during intermediate stages of CKD^10^, it is a major contributor to the increase in cardiovascular mortality by the time patients reach dialysis^23^. Hence, several efforts have been made to reduce established LVH in order to decrease the risk of death. Correction of anemia, intensive renal replacement therapy, blood pressure modification and ultimately kidney transplantation only partially reverse aberrant cardiac remodeling in CKD and ESRD^2, 24, 25^, indicating the existence of other pathologic factors and mechanisms that are specifically activated upon kidney injury and contribute to cardiovascular injury in CKD patients. We have previously shown that FGF23 acts as such an endocrine factor^10^, and here we describe that FGF23/FGFR4-mediated cardiac remodeling is reversible, suggesting its potential for serving as a cardio-protective drug target in CKD. First, removal of FGF23 as a hypertrophic stimulus was sufficient to partially reverse hypertrophic growth of isolated myocytes *in vitro* and to reverse established LVH *in vivo*. Similarly, it has been previously demonstrated that a transgenic mouse model overexpressing a constitutively active FGFR1 mutant form develops a severe cardiac phenotype including LVH, that was in part reversible when transgene expression was discontinued^26^. Second, specific FGFR4 blockade can partially reverse LVH and cardiac fibrosis in 5/6 nephrectomized rats, without ameliorating renal function or affecting severe hypertension. We have previously reported that in this animal model of CKD, FGFR4 inhibition prevents the development of LVH and that pan-FGFR blockade reverses cardiac remodeling^11, 22^. Here, we demonstrate cardio-protective effects of targeting FGFR4, and we show that anti-FGFR4 treatment does not interfere with physiological actions of FGF23 over an extended period of time. In contrast to FGF23 neutralizing antibodies and prolonged pan-FGFR blockade that have been shown to cause hyperphosphatemia, tissue calcification and increased mortality^27, 28^, the specific FGFR4 blocking antibody did not interfere with serum phosphate levels.

We have previously shown that FGF23-induced LVH in mice occurs at fast-onset within only five days of elevating serum levels^10^. Since the hypertrophic response and acute increase in contractility are mediated by FGFR4, we hypothesize that the underlying signaling mechanism driving both events are related. Our findings suggest that FGF23 acutely increases intracellular calcium levels and cardiac contractility, but that sustained activation of cardiac FGFR4 might induce changes that progress to pathologic remodeling including cardiac hypertrophy and fibrosis. Interestingly, blockade of FGFR4 did not rescue the impaired endothelial function mediated by high levels of FGF23 that we had previously observed^21^. This outcome may not be completely unexpected as the effects on the endothelium are mediated by increases of reactive oxygen species and a reduction in NO bioavailability that is likely due to activation of different signaling pathways. These findings indicate that FGFR4 does not mediate all effects of FGF23, including its physiological actions on the kidney that are mainly mediated by FGFR1^29, 30^, and that FGFR4 blockade might not interfere with FGF23-driven vascular pathologies.

Our study of mice on high phosphate diet shows that the reduction of serum FGF23 levels by prolonged administration of high phosphate diet or by switching to normal chow is accompanied by an attenuation of the LVH phenotype but not by a complete reduction of cardiac fibrosis. This is in accordance with our data derived from the 5/6 nephrectomized rats, where FGFR4 blockade reduces LVH but does not significantly affect cardiac fibrosis. Therefore, it is possible that in these models, FGF23/FGFR4 drives hypertrophic growth of cardiac myocytes, as supported by our cell culture data^11^, but might not directly activate cardiac fibroblasts. If true, cardio-protective effects of anti-FGFR4 blockade might be restricted. Interestingly, Hao *et al*. reported pro-fibrotic effects of FGF23 in the heart by targeting cardiac fibroblasts, which mainly occurred in the setting of myocardial infarction^31^. Clearly, future mechanistic studies are needed to elucidate potential direct effects of FGF23 on isolated cardiac fibroblasts as well as in mouse models with tissue-specific deletion of the respective FGFR isoform. Finally, the effects of anti-FGFR4 should be studied in different animal models of CKD, including overall survival, in a time- and dose-dependent manner.

Progressive kidney failure and aging are associated with similar cardiovascular pathologies including LVH that contributes to increased mortality in CKD patients and in the elderly^4, 18^. Human studies have reported that serum FGF23 levels increase with age^20^, and here we show for the first time that FGF23 levels also progressively rise in aging mice with preserved kidney function. Since FGFR4 blockade or deletion in animal models for CKD as well as for aging protects from LVH, we postulate that FGF23/FGFR4 signaling contributes to cardiac remodeling in a wider spectrum of pathologies that do not necessarily involve pre-existing kidney injury. Future animal studies, including the cardiac-specific deletion of FGFR4 in mice, however are needed to determine the direct involvement of cardiac FGFR4.

FGF23 appears to be a novel circulating factor that induces cardiac hypertrophy via activating FGFR4 and subsequent calcineurin/NFAT signaling in cardiac myocytes. Although to date the precise relationship between levels and duration of the FGF23 stimulus and the nature and degree of cardiac injury is still unclear, our current study indicates that FGF23/FGFR4 signaling increases cardiac contractility and hypertrophy in a reversible manner. We postulate that these direct FGF23 actions on the myocardium, at least initially, may have beneficial effects that with increasing serum FGF23 concentrations and exposure time transition into pathological cardiac remodeling leading to a decrease in cardiac function, as observed in animal models with CKD. Although blockade of acute cardiac effects of FGF23 might be not desirable, anti-FGFR4 therapy may protect from chronic cardiac effects of FGF23 and associated progression to irreversible cardiac injury as observed in patients with CKD.

## Material and Methods

### Isolation and culture of NRVM

NRVM were isolated according to our established protocols using a standard isolation system (Worthington Biochemical Corporation). In brief, hearts from 1–3 day old Sprague Dawley rats were harvested, minced and digested with trypsin for 16–20 hours. The next day, trypsin inhibitor was added and the tissue was further digested with collagenase. Cells were released by trituration, filtered twice, incubated for 20 min and pelleted by centrifugation. After resuspension cells were seeded in plating medium [Dulbecco’s Modified Eagle Medium (DMEM; Cellgro) with 17% Media 199 (Invitrogen), 15% fetal bovine serum (FBS; Invitrogen) and 1% penicillin/streptomycin solution (P/S; Invitrogen)] on laminin-coated glass cover slips in 24-well plates. After 72 hours, cells were further cultured in maintenance media [DMEM with 20% Media 199, 1% insulin-transferrin-sodium selenite solution (ITS; Sigma-Aldrich) and 1% P/S] in the presence of 100 μM 5-bromo-2′ deoxyuridine (BrdU; Sigma-Aldrich) for another 4 days and then treated with recombinant murine FGF23 (6His-tagged Tyr25-Val251 [Arg179Gln]; 26.1 kDa) or FGF2 (Ala11-Ser154; 16.2 kDa) (both R&D Systems) at 25 ng/ml for different time points. For treatment studies, an FGFR4 blocking antibody (human monoclonal, U3-11; U3Pharma) was used at 10 μg/ml.

### Immunocytochemistry and morphometry of NRVM

Cultured cardiac myocytes were fixed using 2% paraformaldehyde in 5 mg/ml sucrose for 5 min and permeabilized in 0.3% Triton-X in PBS for 10 min. NRVM were stained for sarcomeric α-actinin (EA-53; Sigma-Aldrich; 1:1000). Cy3-conjugated goat-anti-mouse (Jackson Immuno Research) was used as secondary antibody at 1:300. Nuclei were visualized with 4′,6-diamidino-2-phenylindole (DAPI; 400 ng/ml in PBS for 10 min). Immunofluorescence images were taken on a Leica TCS-SP5 confocal microscope with a 63x oil objective. Myocyte cross-sectional area was measured based on α-actinin-positive staining using Leica AF6000 fluorescence software.

### Contractility measurement in myocardial muscle strips

Twelve-week old wild-type male CD1 mice (Harlan Laboratories; Madison, WI) were euthanized by cervical dislocation. The mouse hearts used for the muscle strip experiments were quickly excised and placed into cardioprotective Ringer’s solution that included the addition of 2,3-butanedione monoxime (30 mM) as described previously^12^. Briefly, left ventricular muscle strips were dissected from the heart in the cardioprotective solution. The ventricular strips were tied on the proximal and distal ends with a silk thread. The muscle strips were then rinsed in Ringer’s solution to remove the 2,3-butanedione monoxime. The muscle strips were hung vertically and attached to force transducer between bipolar platinum stimulating electrodes suspended in 25-ml glass tissue chambers with Ringer’s solution and bubbled under 100% O_2_. Heart muscles were stimulated with pulses of 5 ms duration at 1 Hz and were stretched to the length of maximum force development. Threshold voltage for contraction was identified and final stimulation voltage was set 20% above threshold (approximately 30 V). Muscle strip contractions were allowed to stabilize while media was changed three times over 30 min to wash the strips. A stable baseline was collected prior to treatment and then strips were treated with either vehicle or FGF23 (0.09, 0.9, 9 ng/ml; R&D Systems, Minneapolis, MN). FGFR4 blocking antibody was given at 10.5 µg/ml 30 min prior to FGF23 treatment. Contractile data were recorded and analyzed using LabChart 6 software (AD Instruments, Colorado Springs, CO). Waveform analyses of slope (mN/s), area under the curve (mN × s), and the time constant of decay (rate of relaxation), τ (s), were conducted. Strip experiments were normalized within each condition to baseline levels of contractility and presented as a relative change from baseline contraction data.

### Contractility measurement in thoracic aortic rings

The thoracic aorta of CD1 male mice was rapidly excised and placed in ice-cold HBSS where blood, fat, and excess connective tissues were carefully removed as we have previously described^21^. Briefly, aortic segments 3–4 mm in length were mounted on pins in chambers of a DMT 610 M wire myograph system (Danish Myo Technology A/S, Aarhus N, Denmark) containing Krebs buffer (in mM: 119 NaCl, 4.7 KCl, 0.24 NaHCO_3_, 1.18 KH_2_PO_4_, 1.19 MgSO_4_, 5.5 glucose, and 1.6 CaCl_2_) saturated at 37 °C with a gas mixture containing 20% O_2_/5% CO_2_/75% N_2_ (Airgas Mid South Inc., Tulsa, OK). Arterial rings were progressively stretched to 0.75 g equivalent force passive tension in 0.1 g steps and allowed to equilibrate for 45 min. Aortic rings were exposed to isotonic KCl (40 and 80 mM) to assess the quality of the preparation. To determine endothelial function before performing the experiment, vessels were precontracted with 10 µM PGF_2α_, followed by relaxation response to 1 µM acetylcholine (Ach). Vessels were rinsed with fresh Krebs several times after concentration-response curves. Aortic rings were treated with FGF23 (9 ng/ml) or vehicle for 20 min prior to preconstriction with prostaglandin F2a ﻿(PGF_2α﻿_) (10 µM), and relaxation response to Ach (1 nM to 10 µM) was determined. Aortic rings were pre-incubated with FGFR4 blocking antibody (10.5 µg/ml) for 30 min prior to treatment with FGF23 so as to determine its effect on FGF23-mediated endothelial dysfunction. Force changes were recorded using an ADinstruments (Colorado Springs, CO) PowerLab 4/30 and associated LabChart Pro software (v6.1) running on a standard Windows XP computer platform.

### High phosphate diet in C57BL/6J mice

Twenty 3-month old wild-type C57BL/6J mice (Jackson Laboratories) were switched from normal chow to a 2.0% phosphate diet (Teklad 08020, Harlan). Animals were then randomized in three groups. Six mice were kept on high phosphate for 3 months (PH3/3). Nine mice were kept on high phosphate for a total of 6 months (PH6/6) and 5 animals were kept on a high phosphate for 3 months followed by 3 months of regular chow (PH3/6). Four 9-month old mice on regular chow served as negative controls. Animals were sacrificed and hearts were isolated, perfused *ex vivo* (6 ml of 10% formalin with 4 ml of 20 mM KCl), stored overnight in 10% formalin for fixation, embedded, serially sectioned and stained with H&E and wheat-germ-agglutinin (WGA), as described below.

### Aging of FGFR4^−/−^ mice

FGFR4^−/−^ mice on the C57BL/6J background (kindly provided by Thomas Mariani, University of Rochester Medical Center) were used as a model for global FGFR4 deletion. Six FGFR4^−/−^ mice and 5 wild-type littermates were sacrificed at 18 months of age. Serum was collected and hearts were prepared and analyzed as described above.

### 5/6 nephrectomy rat model of CKD

Chronic kidney disease was induced in Sprague Dawley rats using the 5/6 nephrectomy rat model as described previously^13^. In brief, after mid-line incision the right kidney was surgically removed followed by selective cauterization of two to three branches of the left renal artery. Isoflurane inhalation was used for the induction and maintenance of anesthesia. Postoperative analgesia consisted of buprenorphine injections for 3 days following the surgical procedure. Rats were unbiasedly randomized in the following two groups: 5/6 nephrectomy plus intraperitoneal injections of vehicle (PBS) and 5/6 nephrectomy plus intraperitoneal injections of anti-FGFR4 blocking antibody (U3-11; U3Pharma) at 25 mg/kg/day every 3 days. Treatments started four weeks after surgery. After an additional four weeks, echocardiography and blood pressure measurements were performed, animals were sacrificed and hearts and serum were collected for further analysis.

### Morphology and morphometry of mouse and rat hearts

7 µm short-axis heart sections were stained with H&E (VWR) and used to quantify myocardial thickness as described previously. In short, 7 measurements around the free wall of the left ventricle were taken and averaged. Images were taken on a Nikon SMZ 1500 microscope using 0.75x magnification.

To assess myocyte cross-sectional area, cell borders were visualized with WGA conjugated to Alexa Fluor555 (Invitrogen) according to our established protocols. A Leica TCS-SP5 confocal microscope with a 63x oil objective was used to take immunofluorescence images. We then used Leica AF6000 fluorescence software to quantify cross-sectional area of 25 cells per field at 4 fields along the mid-chamber free wall based on WGA-positive staining.

### Echocardiography of rat hearts

High-resolution echocardiography (15 MHz) in rats was performed at baseline, four and eight weeks after surgery to assess LV mass, wall and chamber dimensions, and systolic and diastolic function using an HDI 5000 Ultrasound system (Philips). M-mode, 2D, and 3D recordings were performed under general anesthesia using continuous isoflurane inhalation delivered through a nose mask while heart rate and body temperature were maintained. Blood pressure was measured in conscious rats using a computerized rattail cuff technique (CODA, Kent Scientific).

### RNA isolation and Quantitative real time PCR

Total RNA was extracted from rat hearts using the PureLink RNA Mini Kit (Ambion by Life Technologies). 2 μg total RNA was reverse-transcribed using First Stand cDNA Synthesis Kit for RT-PCR (Roche Diagnostics). Quantitative PCR reactions were carried out in the StepOne plus Real-Time PCR System (Applied Biosystems) using FAST SYBR Green Master Mix (Applied Biosystems). Raw data were quantified via StepOneTM software v2.3 from Life Technologies. Relative gene expression was normalized to expression levels of β-actin, and evaluated using the 2-ΔΔCt method as described previously^11^.

### Statistical analysis

Data are presented as mean ± SEM. ANOVA and t tests were used for statistical inference with two-tailed p values < 0.05 considered significant. Sample size was not predetermined by a statistical method, but by extensive laboratory experience from previous publications. We did not use formal randomization for any experiment; for *in vivo* experiments, animals were unbiasedly assigned into different groups. Group allocation was not performed in a blinded manner. Whenever possible, experimenters were blinded to the groups (e.g., in immunofluorescence and immunohistochemistry experiments by hiding group designation and genotype of animals until after quantification and analysis). No animals or samples were excluded from data analysis.

### Study approval

All animal protocols and experimental procedures were approved by the Institutional Animal Care and Use Committees at the University of Miami Miller School of Medicine (high-phosphate diet in wild-type mice and aging experiment in wild-type and FGFR4^−/−^ mice), the University of Missouri, Kansas City (contractility measurements of muscle strips and aortic rings isolated from wild-type mice) and the University Hospital Münster (5/6 nephrectomy in rats). All methods were performed in accordance with the relevant guidelines and regulations.

## References

[1] Eckardt K-U (2013). Evolving importance of kidney disease: from subspecialty to global health burden. The Lancet.

[2] Parfrey PS, Lauve M, Latremouille-Viau D, Lefebvre P (2009). Erythropoietin Therapy and Left Ventricular Mass Index in CKD and ESRD Patients: A Meta-Analysis. Clinical Journal of the American Society of Nephrology.

[3] Shlipak MG (2005). Cardiovascular mortality risk in chronic kidney disease: comparison of traditional and novel risk factors. JAMA.

[4] Gutiérrez OM (2009). Fibroblast Growth Factor 23 and Left Ventricular Hypertrophy in Chronic Kidney Disease. Circulation.

[5] Razzaque MS (2009). The FGF23–Klotho axis: endocrine regulation of phosphate homeostasis. Nature Reviews Endocrinology.

[6] Wolf M (2012). Update on fibroblast growth factor 23 in chronic kidney disease. Kidney International.

[7] Gutiérrez OM (2008). Fibroblast growth factor 23 and mortality among patients undergoing hemodialysis. N Engl J Med.

[8] Mirza MAI, Larsson A, Melhus H, Lind L, Larsson TE (2009). Serum intact FGF23 associate with left ventricular mass, hypertrophy and geometry in an elderly population. Atherosclerosis.

[9] Parker BD (2010). The associations of fibroblast growth factor 23 and uncarboxylated matrix Gla protein with mortality in coronary artery disease: the Heart and Soul Study. Ann. Intern. Med..

[10] Faul C (2011). FGF23 induces left ventricular hypertrophy. J. Clin. Invest..

[11] Grabner A (2015). Activation of Cardiac Fibroblast Growth Factor Receptor 4 Causes Left Ventricular Hypertrophy. Cell Metabolism.

[12] Touchberry CD (2013). FGF23 is a novel regulator of intracellular calcium and cardiac contractility in addition to cardiac hypertrophy. AJP: Endocrinology and Metabolism.

[13] Di Marco GS (2011). Cardioprotective effect of calcineurin inhibition in an animal model of renal disease. European Heart Journal.

[14] Molkentin JD (2004). Calcineurin-NFAT signaling regulates the cardiac hypertrophic response in coordination with the MAPKs. Cardiovasc. Res..

[15] Lakatta EG (2003). Arterial and Cardiac Aging: Major Shareholders in Cardiovascular Disease Enterprises: Part II: The Aging Heart in Health: Links to Heart Disease. Circulation.

[16] Dai D-F, Chen T, Johnson SC, Szeto H, Rabinovitch PS (2012). Cardiac Aging: From Molecular Mechanisms to Significance in Human Health and Disease. Antioxidants & Redox Signaling.

[17] Clementi A (2013). Cardiorenal Syndrome Type 4: A Review. Cardiorenal Med.

[18] Andrén B, Lind L, Hedenstierna G, Lithell H (1996). Left ventricular hypertrophy and geometry in a population sample of elderly males. European Heart Journal.

[19] Jovanovich A (2013). Fibroblast growth factor 23, left ventricular mass, and left ventricular hypertrophy in community-dwelling older adults. Atherosclerosis.

[20] Gutiérrez OM, Wolf M, Taylor EN (2011). Fibroblast Growth Factor 23, Cardiovascular Disease Risk Factors, and Phosphorus Intake in the Health Professionals Follow-up Study. Clinical Journal of the American Society of Nephrology.

[21] Silswal N (2014). FGF23 directly impairs endothelium-dependent vasorelaxation by increasing superoxide levels and reducing nitric oxide bioavailability. AJP: Endocrinology and Metabolism.

[22] Di Marco GS (2014). Treatment of established left ventricular hypertrophy with fibroblast growth factor receptor blockade in an animal model of CKD. Nephrology Dialysis Transplantation.

[23] Stack AG, Saran R (2002). Clinical correlates and mortality impact of left ventricular hypertrophy among new ESRD patients in the United States. Am. J. Kidney Dis..

[24] McMahon LP (2004). Development, Prevention, and Potential Reversal of Left Ventricular Hypertrophy in Chronic Kidney Disease. Journal of the American Society of Nephrology.

[25] Rigatto C, Foley RN, Kent GM, Guttmann R, Parfrey PS (2000). Long-term changes in left ventricular hypertrophy after renal transplantation. Transplantation.

[26] Cilvik SN (2013). Fibroblast Growth Factor Receptor 1 Signaling in Adult Cardiomyocytes Increases Contractility and Results in a Hypertrophic Cardiomyopathy. PLoS ONE.

[27] Shalhoub V (2012). FGF23 neutralization improves chronic kidney disease–associated hyperparathyroidism yet increases mortality. J. Clin. Invest..

[28] Yanochko GM (2013). Pan-FGFR Inhibition Leads to Blockade of FGF23 Signaling, Soft Tissue Mineralization, and Cardiovascular Dysfunction. Toxicological Sciences.

[29] Liu S, Vierthaler L, Tang W, Zhou J, Quarles LD (2008). FGFR3 and FGFR4 Do not Mediate Renal Effects of FGF23. Journal of the American Society of Nephrology.

[30] Gattineni J (2014). Regulation of renal phosphate transport by FGF23 is mediated by FGFR1 and FGFR4. AJP: Renal Physiology.

[31] Hao H (2016). FGF23 promotes myocardial fibrosis in mice through activation of β-catenin. Oncotarget.

